# “*I feel so bad but have nothing to do.”* Exploring Ugandan caregivers’ experiences of parenting a child with severe malaria and subsequent repeated uncomplicated malaria

**DOI:** 10.1186/s12936-018-2514-z

**Published:** 2018-10-12

**Authors:** Ann J. Nakitende, Paul Bangirana, Noeline Nakasujja, Margaret Semrud-Clikeman, Andrew S. Ssemata, Chandy C. John, Richard Idro

**Affiliations:** 1 Department of Psychiatry, College of Health Sciences, Makerere University, P.O. Box 7072, Kampala, Uganda; 2 Department of Pediatrics, University of Minnesota, Minneapolis, MN USA; 3 Department of Pediatrics, Indiana University, Indianapolis, IN USA; 4 Department of Pediatrics and Child Health, Makerere University, Kampala, Uganda; 5 Centre of Tropical Medicine and Global Health, University of Oxford, Oxford, UK

**Keywords:** Repeated malaria attacks, Caregivers, Children, Severe malaria, Uncomplicated malaria, Parenting

## Abstract

**Background:**

Severe malaria in children is often associated with long-term behavioural and cognitive problems. A sizeable minority of children go on to experience repeated malaria due to the high transmission and infection rates in the region. The purpose of this study was to explore caregivers’ experiences of parenting a child with a history of severe malaria followed by repeated episodes of uncomplicated malaria in comparison to healthy community children.

**Methods:**

Thirty-one caregivers were enrolled in the study. These included caregivers of children previously exposed to severe malaria and who had experienced repeated uncomplicated malaria attacks (SM with RMA, n = 15), caregivers of children exposed to severe malaria who did not experience repeated episodes (SM, n = 10), and caregivers of healthy community children (CC, n = 6) were purposively selected.

**Results:**

Thematic-content analysis generated eight areas of concern, six of which were noted only by caregivers of children with SM or SM with RMA: (1) a sense of helplessness; (2) challenges with changes in behaviour; (3) responses to a child’s behaviour; (4) family life disruptions, including breakdown of relationships and inadequate male-spouse involvement in child care; (5) disagreements in seeking healthcare; (6) societal burden; and two by caregivers of children with SM, SM with RMA and also CC; (7) concern about academic achievement; and, (8) balancing work and family life.

**Conclusions:**

The study findings suggest that severe malaria, especially when followed by repeated malaria episodes, affects not only children who have the illness but also their caregivers. The effects on caregivers can decrease their social functioning and isolate them from other parents and may disrupt families. Interventions to support caregivers by counselling the ongoing problems that might be expected in children who have had severe malaria and repeated episodes of malaria, and how to manage these problems, may provide a way to improve behavioural and mental health outcomes for those children and their caregivers.

## Background

Parenting is a process of interactions and relationships that nourish, protect and guide each new life through its course and development [1]. Parents and community members are filled with joy and excitement at the birth of a child [2]. This phase of parenthood brings adjustments and responsibilities to nurture a child from birth to a self-reliant adult [1]. As children grow, the parenting role may be challenged by worry about a child’s frequent hospitalizations to deal with the *sequelae* of disease and medical expenses [3]. Previous research has evaluated the responsibilities faced in parenting children with chronic conditions, such as asthma [4], sickle cell [5], cerebral palsy [6], cancer [7], intellectual disability [8], mental illness, traumatic brain injury [9], and nodding syndrome [10]. Other than their everyday living challenges, caregivers to normal children also experience shortcomings in availing necessary resources.

Malaria presents a huge public health challenge globally, with an annual estimate of 216 million clinical cases and 445,000 deaths, despite commendable strides in prevention and control [11]. Of the global annual malarial infections, 88% are in Africa with the sub-Saharan region being the most affected. Approximately 90% of deaths due to malaria occur in the sub-Saharan region and an estimated 70% are children under 5 years of age [11]. Although globally there has been an approximate 65% decline in mortality among children [12], in Uganda malaria continues to be the leading cause of morbidity and mortality with a reported 27.2% of inpatient deaths among children aged under 5 years [13]. Of concern is the increase in repeated malaria episodes that are reported both from outpatient visits and inpatient hospital departments [14]. Indeed, African children experience between 1.6 and 5.4 episodes of malaria each year [15]. Uganda, in addition to Cambodia, Djibouti, Madagascar, and Venezuela, has the highest number of cases of repeated malaria attacks (RMA) due to *Plasmodium falciparum* [16].

*Plasmodium falciparum* is responsible for the most severe and repeated form of malaria [16]. Severe or complicated malaria (SM) is associated with increased mortality and may present with anaemia, impaired consciousness, low blood glucose, prostration and repeated convulsions [16]. Even in less fatal form, malaria may affect the brain and some children suffering from severe forms develop long-term cognitive and behavioural problems [17–19]. Due to behavioural problems exhibited in some children with a history of SM [19, 20], caregivers may seek assistance from other family members to take care of the siblings, placing higher demands on the whole family. Socio-cultural issues have been reported by caregivers as barriers to prompt treatment of childhood malaria, and failure to differentiate a natural illness from traditional illness in seeking treatment for SM among children [21, 22]. In a Ugandan exploratory study about caregivers’ treatment-seeking behaviour for febrile children aged 5 years and under, with presumed malaria, caregivers reported challenges in lack of knowledge in administering appropriate malaria treatment for their children and financial medical constraints in managing negative malaria episode outcomes [23]. Within these studies caregivers were found to experience psychological, marital, social, and economic challenges. Understanding the experiences that a caregiver faces in caring for a child with SM or SM and RMA besides administering anti-malarials, is pertinent. There is a dearth of information on caregivers’ experiences in parenting a child with RMA and no known study to date has evaluated these experiences in a Ugandan population. The purpose of this study was to explore caregivers’ experiences of parenting a child with a history of SM and RMA.

## Methods

### Study description

Qualitative methods were used to explore caregivers’ experiences in parenting children with a history of SM, RMA and CC. This study was nested in a prospective cohort study conducted at Mulago Hospital, Kampala, Uganda recruiting children between 18 months and 12 years old. Details of the study procedures are described elsewhere [17, 20].

### Instrument

Caregivers’ demographic characteristics were recorded on a standardized sociodemographic questionnaire. AJN formulated questions in relation to the research question. Discussion meetings with the co- authors PB, ASS, NN and RI were conducted to ensure that the interview guide had simple, open-ended, unambiguous questions appropriately set to capture caregivers’ descriptions. From these discussions, the number of questions was reduced from 27 to 8 key questions that were developed and consolidated to form the final interview guide. Probe questions were posed for further descriptions. The interview guide was translated into Luganda and English, respectively, to ensure credibility and trustworthiness of the findings. The interview guide was used to collect information on: behaviour before and after SM, behaviour after RMA; difference in behaviour from other children in the household and community; perception of community members of a child that suffered from SM and RMA; how the caregivers managed the child; relationship with family members; prospects of childbearing; and, treatment options.

### Participant recruitment

Caregivers were purposively selected between July and September 2015 from the prospective cohort study registry. A child’s identification and caregiver contact number were obtained from the registry and the caregiver contacted through telephone calls and invited to attend a 40- to 60-min interview. The purpose of the study was explained to the caregiver. Confidentiality and voluntariness to participate were emphasized. Clarification on any raised concerns before the interviews were made and respondents were assured of anonymity of their identity. In-depth interviews were used to obtain caregivers’ subjective opinions about experiences in parenting a child with a history of SM and RMA and those without RMA inclusive of CC. The CC children were identified from the prospective cohort study. CC children were defined children from the nuclear family, neighbourhood or external household of children with CM or SMA who did not have chronic illness, acute illness at the time of enrolment, major abnormalities on screening history or physical examination, developmental delay, or past history of coma. A child was said to have RMA if from the registry, they presented with a positive blood smear for malaria on 3 or more occasions at least 4 months post discharge. None of the caregivers that consented to participate in the interviews withdrew consent or opted out. A total of 31 in-depth interviews were conducted. The respondents’ ages ranged from 23 to 65 years. In-depth interviews were conducted by AJN and ASS, both psychologists with training in qualitative research methods. The in-depth interviews were conducted either in a quiet clinical room or in respondents’ home environment. Caregivers were interviewed till a point of saturation where no new information emerged. In-depth interviews were audio-recorded, while notes were taken for two caregivers who declined to be recorded. Of the 31 caregivers, 15 had children with a history of SM and RMA, 10 had children with SM and 6 were CC.

### Ethical approval

The study was approved by Makerere University School of Medicine Research Ethics Committee and the Uganda National Council for Science and Technology. All participating caregivers provided written informed consent.

### Quality assurance

Prior to data collection, the interview guide was pre-tested on two caregivers who had children that had not suffered SM in the prospective cohort study. The pre-test was conducted to ensure that closed-ended questions such as ‘Are you related to this child?” were rephrased to open-ended queries: “How are you related to this child?” while some questions that did not capture the objective of the study were discarded. Ambiguity was modified to check for appropriateness of the questions and to ensure the questions were precise and understandable, and questions that did not allow the respondent to express at depth had prompts attached. During the pre-test the reasonable time to conduct the interview was noted. The pre-test enabled the interview guide to capture questions more sequentially and logically in line with the objective of the study. For the two caregivers who declined an audio recorder, the coder interviewed while notes were taken.

### Data management and analysis

Thirty-one in-depth interviews were conducted; 29 in-depth interviews were audio recorded, transcribed verbatim and those recorded in the local language were translated into English. These interviews were typed in a Microsoft Word document. Thematic-content analysis was used because it allows for identification, interpretation and comparisons between concepts across diverse respondents [24]. The analysis started with manual coding of the transcripts. The transcripts were read independently several times to become familiar and search for similar and divergent patterns in the quotes. The different generated codes were later merged to form themes. The identified themes from the data were written on the right-hand side margin of the transcripts. All themes with similar thread were clustered together in a coding matrix. A main theme was then obtained for each cluster of themes. Relevant verbatim quotations obtained from the 31 in-depth interviews were reported in the analysis and anonymity of caregivers’ and children’s identity ensured.

## Results

Of the 31 respondents, 26 (84%) were female with a median age of 44.0 (age range 23–65 years). Male respondents’ median age was 39.0 (age range 32–46 years). The majority of female respondents were married or living with a partner. Fifty-two per cent of respondents had attained primary education level. Table 1 provides demographic characteristic of caregivers.

**Table 1:** Demographic characteristics of caregivers

Respondent code	Type of caregiver	Gender	Age	Marital status	Educational level	Employment status
1	Father	M	32	Single	Tertiary	Farmer, boda boda rider
2	Mother	F	35	Married	P.7	Sells food stuff
3	Mother	F	32	Married	None	Unemployed
4	Father	M	40	Married	P.7	Sells food stuffs like beans
5	Mother	F	41	Separated	P.2	Digs for people in the village, does any job
6	Father	M	37	Married	P.4	Builder
7	Grandmother	F	65	Married	P.7	Farmer
8	Mother	F	31	Married	P.7	Sells food stuffs
9	Mother	F	30	Married	P.6	Businesswoman
10	Mother	F	30	Married	Secondary	Washes clothes
11	Mother	F	29	Married	P.2	Unemployed
12	Mother	F	24	Married	Secondary	Sells food stuff
13	Mother	F	35	Widow	Secondary	Sells food stuff
14	Mother	F	36	Married	Secondary	Businesswoman
15	Mother	F	36	Separated	None	Unemployed
16	Mother	F	36	Married	Secondary	Businesswoman
17	Grandmother	F	55	Single	P.3	Sells handicrafts
18	Mother	F	30	Separated	Secondary	Sells charcoal
19	Mother	F	23	Separated	P.5	Unemployed
20	Grandmother	F	60	Separated	None	Unemployed
21	Mother	F	28	Married	P.6	Tailor
22	Father	M	46	Married	Tertiary	Carpenter
23	Mother	F	40	Married	Secondary	Farmer
24	Father	M	34	Married	P.4	Farmer
25	Mother	F	39	Single	P.6	Farmer and rears fowls and cattle
26	Mother	F	41	Married	P.6	Unemployed
27	Mother	F	32	Married	P.5	Unemployed
28	Grandmother	F	60	Married	None	Sells doughnuts “mandazi”
29	Mother	F	39	Married	P.7	Sells food crops
30	Mother	F	31	Separated	Tertiary	Saloon
31	Mother	F	50	Separated	Secondary	Farmer and businesswoman

P means primary level

The study findings represent caregivers’ experiences of parenting a child with SM or SM with RMA and CC based on eight major thematic areas of concern, presented with excerpts that emerged from the annotated caregiver transcripts. Table 2 summarizes the thematic areas of concern and the disease groups (SM, SM with RMA or CC) in which caregivers noted these concerns.

**Table 2:** Thematic areas of concern expressed by caregivers of children with severe malaria (SM), severe malaria with repeated subsequent malaria attacks (SM + RMA) or otherwise healthy community children without any history of severe malaria or repeated malaria episodes (CC)

Area of concern	Clinical group		
	SM	SM + RMA	CC
Sense of helplessness	X	X	
Changes in behavior	X	X	
Academic achievement	X	X	X
Caregiver response to child’s behavior	X	X	
Family life disruptions	X	X	
Balancing work and family life	X	X	X
Disagreements in seeking health care	X	X	
Societal burden	X	X	

### Sense of helplessness

The caregivers expressed a great sense of helplessness and in some cases despair in managing a child who was suffering from SM or SM with RMA. They expressed fear, worry and uncertainty of what the future holds for their children:

“*Ok. One thing I had to understand and know was that I have to live with him, look after him and work very hard… You know when you are “*oli mubanga” *in ‘space’ (helpless) you tend to worry and fear so much and I end up not doing what I am supposed to do, like work.*” (ID 1)

“*I feel so bad but have nothing to do about it. My worry is she is getting older and not studying. Soon the years are going by.*” (ID 11*)*

Due to past experiences of a child’s SM, caregivers felt uncomfortable and unable to manage subsequent situations when their children suffered from RMA. They perceived themselves as powerless and made appeals to anyone to offer them solutions.

“*Ok I would like the healthcare providers to have a programme say on radio to help us understand or teach us how we should ‘treat’ these children not to suffer from repeated malaria; how to deal with it.*” (ID 3)

Although some seemed powerless, other caregivers of children who suffered SM with RMA who found themselves in similar situations sought spiritual and traditional healing to cope with the difficult situation:

“*I was hurt and called out to God; whenever she gets malaria I feel distressed (*ennaku*) and call on God; and I surrender all to God to take charge of the situation; but I worry so so so much; and people say these kind of children don’t live long.*” *(*ID 8)

“*Yes at times I would get* “ebombo” *(traditional herb) and give them but I got tired so I resorted to praying in faith even as I give them medicine from the hospital*.” (ID 16)

This theme was expressed among the caregivers of children who survived SM or SM with RMA but not the CC children.

### Challenges with changes in behaviour

Often when a child suffered from SM or SM and subsequent RMA, caregivers reported observing behaviour changes in their children:

“*When he started suffering from the repeated malaria, he became sadder (*yenakuwaza nnyo kati*), does not play with friends even when encouraged, becomes angry; when you call on him to participate in an interesting game where you are, he only looks at you in anger, gets quiet, or sleeps or decides to take on sole play.… when he asks for money and you don’t provide it, he will go to the shop and ask for something, and tells the shopkeeper that* ‘maama’ *(mother) will bring the money later; so you are told to pay when you pass by the shop.*” (ID 5)

A caregiver whose child suffered SM and RMA showed fearless behaviour:

“*He is fond of handling and touching things that kill. Behaviour started after falling sick again… I asked him what he needed the medicine for and he said he needed to drink to die. He spends most of his time just there; he often picks broken bottles from the rubbish pit and checks for its contents… I see him with razorblades,… he doesn’t fear snakes. There is a time when I heard him shouting and laughing so hard; so I wondered what had amused him; so when he saw me, he told me to walk slowly not to frighten his animal away; I asked and he refused to show me where the animal was; he later showed me where the snake was. I shouted while calling him out of harm’s way but the child refused to leave the place where the snake was. People came and helped me kill it. After it had been killed, he held it by the tail and dragged it; you could actually see a child who didn’t have fear for dangerous animals*.” (ID 3)

A caregiver to a child with a history of SM expressed changed slowed thought process:

“*She is different in thinking if you send her for something she goes to the house and comes back and asks you what did you send me for? It came after she had fallen sick because before she would do the house work before you even tell her she would even tell her siblings what to do but now when she is playing you have to tell her do like this but even then she continues playing till you have to shout at her again.*” (ID 22)

Compared to caregivers of children who had suffered from SM or SM with RMA, caregivers of CC felt they had achieved age-appropriate developmental milestones, and did not express concern about abnormal behaviour:

“*His behaviour is not any different. These children all tend to be stubborn* balinamu eddalu *… they fight and then play like that just like any other children. I do not see any difference.*” (ID 29)

### Challenges in academic achievement

From the discussions, caregivers of children with SM or SM with RMA and CC all expressed worry and uncertainty of what the future held for their children in terms of achieving in school:

“*Her knowledge (*amagezi*) reduced. Before she would study and I could see she understands what she is reading but now I see that what she does is not that good. She is defeated she can’t read.*” (ID 25)

“*In her studies before she could recite from 1 to 20 but after she fell sick her brain was distorted she finds it hard to count.*” (ID 22)

A child’s ability to achieve in school may often be affected by SM and SMA, for example, once not guided and supported by their caregivers, schooling becomes difficult:

“*His mother has been very involved. She chopped sticks and stones, to help him to learn how to count and write the numbers; though writing his name is still a challenge.*” (ID 11)

From the interviews, it was noted that challenges in academic achievement not only affected children with RMA, as some caregivers of CC reported speaking with class teachers to find solutions to academic problems:

“*I also visit these children at school and speak with the teachers about class work and learning. We try to rectify what has failed gradually.*” (ID 27)

### Caregiver response to child behaviour

A child’s behaviour can frustrate a parent who may resort to beating their child because the parent believes the child is deliberately misbehaving. This was noted for children surviving SM or SM with RMA but not among CC:

“*Ehh, there is a time when I slapped him because it was too much for me to tolerate his behaviour and wondered that probably if I didn’t punish him, he might continue to deliberately behave that way.*” (ID 3)

“*Ok I don’t become happy (*sisanyuka*), but from the time that they explained that this illness does sometime change a child so sometimes I would not beat him, I handle him softly but sometimes I realize that it’s too much at least he is somehow improved I beat him in order to achieve sustainable behaviour change (*obutalemera yo*) not to become stupid (*okusiliwala*) and not to consider himself as a person who shouldn’t work. At first ‘*namukwata bulungi’ *(handled him gently).*” (ID 10)

Children who suffer from SM and RMA may often either regress in developmental milestones or exhibit changes in behaviour that caregivers perceive as deliberate to provoke punishments. Caregivers culturally take on a “spare the rod and spoil the child” stance in disciplining a child. This prevents the caregiver from regretting not having shaped appropriate behaviour earlier on in the child’s development. Some caregivers reported shouting at the child to make them realize and change their inappropriate behaviour:

“*I would beat her, shout at her and tell her “you want to become stupid”. Then I realized that I had to change because I was her mother I needed to treat her differently so that she would not fear me and besides me who would take care of her? Only me.*” (ID 2)

### Family life disruptions, breakdown of relationships and inadequate male-spouse involvement in child care

This theme presents varying family dynamics among children that suffered SM or SM with RMA. When a child repeatedly falls sick, with no apparent understanding by one or both parents of the cause of the illness, this causes a rift between parents. In many contexts, the father is particularly affected due to financial burden of medical bills and cost of prescribed medicine that they have to bear in a patrilineal culture such as Uganda:

“*The father of the child would just say what is wrong with this child who keeps falling sick without getting well and there is no money. He used to wake up and go, and one day he never returned.*” (ID 25)

“*So this has affected the children very much he was not providing food so I had to go work to see that the children are well so after some time we stopped seeing him at home.*” (ID 21)

“*It is now a year since we separated. He is not interested even when I call him.*” (ID 18)

Due to a child repeatedly falling sick, changes in family dynamics and breakdown of relationships frequently occur. From the discussions, it was clear that some fathers became unconcerned and uninvolved in childcare. A failure to understand why a child suffers RMA emotionally and financially strains caregivers, leading to a breakdown in the family.

The attitude of the fathers for some of the girls compared to the boys who suffer RMA was worse:

“*Her father has many wives; he says why is it that she is the only one among all children that is falling sick. After all she is a girl, he says if she doesn’t pass primary one he is not going to waste money on a stupid child.*” (ID 2)

In some relationships although the husband may not fully support, he sympathizes with and advises the mother to care for the child even though he may not provide the transport fare or money to buy medicine.

“*He tells you to take the child to hospital; when you call him, he comes; but still he doesn’t pay for the medical care; you have to involve yourself fully to meet the cost so that the full dose is given; otherwise he doesn’t.*” (ID 5)

Some fathers were reported to express strong emotions and feelings about responsibility of caring for their children that suffered SM:

“*I can’t leave her with anyone because they don’t know how to take care of her, but also her father refuses to have her go off because he says they may not take good care of her like we do. There is even a time* jajja *(grand*-*parent) came from Mbale (a countryside town) and asked to go with her to the village for the holiday but he refused and said unless she goes with her mother.*” (ID 19)

Caregivers expressed varying views about future prospects of childbearing which is a key social issue. Caregivers expressed the need to postpone childbearing to adequately care for an ailing child:

“*Ehh I completely do not want to give birth again, considering what I have gone through with this child; I fear that the situation may be the same in case I gave birth again; it hasn’t been easy looking after this sickly child.*” (ID 5)

On the other hand, some caregivers perceived the dependent child as of no use in future and therefore they needed to give birth to another child as replacement:

“*I need to give birth again not now but later just in case my child doesn’t heal. I need to have another healthy child.*” (ID 2)

For CC, in contrast, these concerns were not voiced, and fathers to CC were more involved in childcare, especially the healthcare of their children, and were attentive to financial needs of the entire family:

“*His father looks after him. He cares for him entirely and provides for him. When you tell him about his needs, he provides.*” (ID 28)

“*He looks after the entire family and provides for us financially.*” (ID 26)

### Balancing work and family life

Caregivers to children with a history of SM or SM with RMA expressed inability to continue attending to their work and at times had to leave their job to attend to their sick child, which reduced the family income. In other instances caregivers were faced with the challenge of carrying the child along to work in order to fulfil the financial obligations for the family:

“*It affects my work so much. Because when he falls sick I don’t work. Whenever he falls sick, I don’t do any job.*” (ID 1)

“*I had to leave work and take care of the children. I was working in town selling children’s clothes but I left. After the children had recovered I got money and started selling charcoal from home and I also bought some pigs.*” (ID 16)

While some caregivers reported an inability to continue with work, some of those who had left work to look after their children were able to find or start up income-generating activities at home and work from home in order to support the family. Others searched for ways to make money (i.e., washing clothes for other people, tailoring at home), which does not provide as much money as working away from home:

“*I help my sister sell clothes in the mobile markets…when he fell sick, I would not leave him under the care of anyone; I had to place a small shop at home; after he recovered, I resumed work. It affected me because at home you can’t earn that much money like when you hawk the items.*” (ID 14)

Balancing work and family life seemed to be a concept shared by caregivers to CC, not based on a child’s health particularly, but on overall growth and development. Some caregivers agreed with their spouses to leave their job, to holistically take care of the children:

“*Their father said if I take on a job, these children will not perform well in school because they will often miss being at home, they might find the food not yet prepared and clothes unwashed. So he decided that I should first stay at home as the children grow so that when one of them reached secondary school, she can return home and do house chores.*” (ID 26)

### Disagreements in seeking healthcare

Caregivers reported challenges in decision-making and disagreements with family and some community members as to where they should take their sick child for further healthcare. This was not expressed among caregivers of CC:

“*I was in Mulago (a referral hospital), they would call everyday telling me; ‘take him out of hospital you are going to be the cause of your child’s death, that is* ‘olumbe o’luganda’ *(traditional illness); so I insisted on not taking him out of Mulago saying this is* ‘olumbe o’luzungu’ *(illness that can be treated using modern medicine). I asked them who can bewitch this young child. They insisted that we should leave hospital otherwise my son is dead; even my siblings were telling me to leave the hospital. They all said that was* ‘olumbe o’luganda’*. I insisted on staying in Mulago.*” (ID 3)

“*They thought probably satanic causes. I too thought of the same; I was in agreement with them for why is it that my child is the only one in the area who often fell sick; and sometimes when you have earned and saved some money, that is when he would fall sick and all the saved money is spent on his treatment; I thought hard about it but I didn’t take him there (to traditional healers).*” (ID 10)

Because of the beliefs expressed, the caregiver needed to reconsider and take on a personal decision to the treatment provided for a child who has repeated ailments that do not seem to respond to medical treatment. Cultural beliefs influence the ability to seek healthcare in either the western, spiritual or traditional sphere. The caregiver is blamed for going against majority decision. Some caregivers to children with a history of SM or SM with RMA reported seeking western healthcare first because of easy accessibility to the national healthcare facility.

### Societal burden

For some caregivers, their community members perceived children with a history of SM with RMA as a burden whenever there was re-occurrence of RMA. Caregivers of children with SM and RMA reported situations where they had to borrow money or medical supplies from community members and clinics to take care of their unwell children. However, before they even paid back, the children were taken ill again, thus a failure to pay back and a need to borrow more:

“*Some community members give me monetary loans to see that I treat her. Other people give me food to feed her. At the clinic I can ask them to treat her on loan then I pay later when I get the money.*” (ID 4)

In other situations, caregivers had to leave a child under the care of a neighbour to execute other family obligations. The neighbour who may be engaged in her or his own duties may not execute the child-caring role adequately nor have a clear understanding of the child’s needs. Caregivers are emotionally involved and feel that only they can best understand their child’s behaviour:

“*I left him there one time, I found when he had wandered off in the same path that I had taken; someone picked him and took him back. So instead of leaving him at someone else’s place, I would rather leave him outside standing at the door. I feel bad but I have no alternative. Ok most of the time when I am to travel a long journey, I carry him along to prevent any problems; and not to worry too much; that maybe I will find him beaten after annoying someone; which may lead him to faint; so most of the time, I travel with him.*” (ID 3)

## Discussion

The findings of this study indicated that caregivers of children with a history of SM and those of children with SM and RMA both experience emotional, psychological, social, and economic challenges; caregivers of children who suffered SM with RMA experienced the most.

Changes in behaviour were reported by some caregivers to have manifested after a SM episode while others were able to note that the change in behaviour was after SM with RMA. Behaviour described after RMA included sadness, startling, solitude, picking things at the shop without caregiver’s permission, showing no fear for harmful animals, anger outburst, lying, fighting with siblings and peers, attempts to wander away from home if not restrained and not paying attention to given instructions and failure to execute tasks to completion. These behaviours are similar to those caregivers reported among post-exposed cerebral malaria children attending a paediatric neurology clinic in Malawi, who acted aggressively towards friends, were destructive and short tempered [25]. Similarly, studies done in Uganda among children with SM in comparison to health CC reported aggression, outbursts of anger, attention difficulties, impulsivity and crying spells [19, 20, 25, 26]. In contrast to the current study, some of these studies used standardized and diagnostic measures to assess behavioural difficulties, limiting a caregiver’s description of behavioural events. The study was able to subjectively explore experiences and behaviours from the caregivers. Caregivers reported executing different disciplinary actions that involved verbal or physical punishment for the abnormal behaviour, sometimes thinking the children were deliberate in their behaviour. From these interviews it became evident that there is a need for education of parents as well as psychological interventions for children that have suffered adversity due to SM or SM with RMA to prevent mental health problems later in a child’s development. In addition, healthcare professionals need to continuously provide malaria health education to enhance caregivers’ knowledge about malaria, its causes, transmission, and protective measures to prevent RMA and alleviate any behavioural and emotional changes during the child’s development.

Challenges in academic achievement were expressed by the caregivers in all three groups of children. However, the challenges were expressed more by caregivers of children who suffered RMA. Caregivers reported school absenteeism whenever a child suffered from RMA, which affects a child’s academic achievement. The study findings relate to studies done in Sri Lanka [27] and Uganda [28], where children’s attendance and school attainment improved only after treatment on anti-malarial prophylaxis compared to the placebo group. These were randomized, controlled studies that employed quantitative methodologies which differs from the design used in this study. These studies do not make mention of how caregivers were involved in improving children’s school attainment. Noteworthy in this study, while some caregivers reported no change in academic achievement, there were suggestions of improvement in academic achievement after continuous and repeated caregiver-assisted tutoring to children with a history of SM with RMA. Caregivers to CC indicated liaising with class teachers to find solutions for better performance. Furthermore caregivers showed strong enthusiasm in stimulating their children through repeated engagements and motivation to enhance achievement in school through using environmentally friendly teaching guides. Although caregivers expressed emotional distress over their children facing difficulties in school work, they established measures to ensure their children attained some basic school skills. Communication has a central role in the academic achievement of a child where there is need for interaction between the school teacher, peers and neighbours [29]. The more closely interrelated in their level of communication and socialization is, the more likely this will improve a child’s academic achievement and interpersonal relationships. A child’s ability to achieve in school will depend on the quality of instruction and interaction a caregiver has with teachers and a caregiver’s value of academic achievement [30].

Family life disruptions, breakdown of relationships and inadequate male-spouse involvement in childcare are other concerns that emerged from the interviews. Whereas some fathers left their families, others were reported to provide care for their children. More surprising, findings in this study indicate a shift in fathers having to abandon their families and their failure to take on the responsibility to provide finances for their children’s healthcare when they suffered from RMA. Being a patriarchal society, it is expected that a father takes on the requirements of a head of the family by providing financial and emotional support to the family. When family members are emotionally close, this increases spouses’ mutual understanding and positive child development [31]. Marital distress not only affects either spouse caring for a child but also impacts the child’s development when he or she perceives the family environment as insecure, which may lead to feelings of low self-esteem and rejection [32]. The findings are in agreement with Ambikile and Outwater [33], where parents expressed lack of spousal support and social life problems that affect child development. In contrast, for families where the father was present, such as in CC, mutual commitment was observed in steady provision of resources for family members. This promotes communication and timely problem solving when making decisions on parenting. The study findings show how a child’s ill health may help bond the dyad relationship that leads to sensitive and responsive care which will promote a child’s ability to perceive a secure environment. This creates either adaptive behaviour later in a child’s life or disruption of interpersonal relationships within the family leading to different expression of emotional reactions indirectly to the child and among caregivers. When spouses are not present or helpful, the situation may impact on the interaction within family members, putting the primary caregiver at risk of depression. This finding shows the need for social support among caregivers when faced with challenges of caring for children with RMA, particularly when one partner is absent or unsupportive. The events at home can affect progress of a child’s development and potential to achieve later in life. Further, this finding shows that a mother’s ability to deal with her child depends on social support received from friends, neighbours and relatives who play a part in her ability to effectively care for her child who suffers from RMA.

Furthermore, findings in this study indicate the effect that children with RMA have on the socio-economic status of their caregivers. The *sequelae* of the disease frequently leads to changes in work environment, the type of work that can be accomplished or in leaving a job to care for the sick child without the ability to earn money to provide for family and healthcare needs. The social-economic equilibrium in the family is affected when a child suffers from SM or SM with RMA [34]. This indirectly affects the family’s financial progress since money is needed to purchase medicines and transport an ailing child to a healthcare facility whenever they suffer a RMA. This finding is similar to a study exploring parents’ experiences of caring for a child with autism spectrum disorders [35]. This disease affects family members as well as burdening other society members from whom food stuff and money is borrowed and may not be paid back in time. Consequently, the socio-economic status of the caregiver exerts an indirect influence on the child’s development. A child’s emotional relationships are affected by a caregiver’s ability to earn, in order to adequately provide for the needs and healthcare whenever a child suffers from RMA.

One of the consequences of the illness is that in the long run these children may not be productive members of their community and their illness saps their caregiver’s ability to earn a living. The children’s productivity may indirectly be affected by the effect the illness has on their physiological growth, and educational disruptions, including frequent school absenteeism due to RMA, may affect their future performance and progression and have long-term impact on earnings [36, 37]. SM and RMA, in the long term, indirectly affects the economic productivity of a country [34]. Further, Nabyonga Orem, Mugisha [3] in their study of health-seeking patterns and determinants of out-of-pocket expenditure on malaria in children under 5 years in Uganda, purport that caregivers need money to access healthcare, which money is not always available. The study findings show the need for financial interventions, to provide education for fiscal management including income-generating activities at home from where they can generate a cash flow, to provide for household and healthcare necessities while caring for their ill child.

The study findings revealed that caregivers did practice traditional and spiritual healing alternatives, as opposed to western health remedies, for the re-occurrence of RMA which caregivers neither understand nor have an explanation for. Caregivers with strongly held cultural beliefs explain to themselves and their communities that the repeated and chronic diseases are due to supernatural and spiritual causes [38]. Family members and community may blame the caregiver for having misbehaved and annoyed the spiritual world which causes their child’s frequent sickness [33]. This positions the caregiver as one who needs to “appease the gods” for the child’s wellbeing and also put an end to the re-occurrence of RMA. This situation creates conflict when a caregiver receives disapproval from within the family or the community and creates a state of cognitive dissonance as to where to seek healthcare. It also places a burden on the caregiver to be the one to take on sole initiative to decide on treatment options for their child whenever they suffer RMA. Similarly other studies have found traditional beliefs influence where caregivers seek treatment for malaria in children aged under 5 years [23]. The inability of a caregiver to make prompt decisions to seek healthcare may cause more adversity to the psychological and physical development of a child and possibly worsen the child’s medical situation. These experiences influence the interaction of how the family and society perceive and relate to a child that is often ailing, whereas different caregivers may perceive these experiences differently. The interaction and how a child relates in behavioural and cognitive processes may be impacted by SM and RMA and the relationship with the caregiver.

## Limitations of the study

While the design consisted of a sample of 31 respondents, their experiences cannot be generalized to all caregivers who are involved in parenting children with SM and RMA. Although some males were interviewed, this was not sufficient to show the male perspective. Despite the limitation, of insufficient male participation, the researchers were able to compare caregivers in the study to a control group to provide information as to whether the experiences expressed in context differ or are similar. For further research directions, these findings can be a base to conduct a quantitative study with more participants to assess caregivers’ experiences in caring for children with RMA and their coping strategies.

## Conclusions

This is the first study to provide an understanding and description of caregivers’ experiences in parenting a child with or without a history of SM and RMA. Caregivers experienced child-related and personal issues related to family disruptions. These disruptions involved the breakdown of relationships and inadequate male-spouse involvement in childcare, a sense of helplessness in the management of a child with SM and RMA, an inability to work, the societal burden, and disagreements in seeking healthcare and challenges with a child’s changes in behaviour and achievement in school. Findings from this study further describe how caregivers of children with RMA have their social functioning disrupted by a lack of “me time” to rejuvenate their emotional energy for a better psychological wellbeing. These caregivers worry about their children’s health as well as the daily stressful disturbances of visits to the healthcare facilities or hospitalization that was not expressed among caregivers to healthy children.

An important point from this study is the impact a child’s illness has on the social and economic aspects of the family. The families of children who suffer from RMA frequently experience significant problems with financial support, husband abandonment, lack of financial and emotional distress from the husband, and a lowered standard of living as the mother needs to seek employment at home. If these challenges are not addressed early on, the psychological development of a child may be affected, resulting in later life adversity. For these reasons, there is a need for early interventions that support the caregiver, encourage healthy discussion between parents, and provide guidance for caregiver-child interactions for caregivers of children who have severe malaria, particularly SM with RMA, and for the children themselves. Such interventions, though admittedly complex to develop and administer, could potentially prevent later mental health problems for the child, and improve mental health and day-to-day function for caregivers by providing skills to ameliorate the physical and emotional demands that these caregivers face. There is need for policy makers to ensure implementation and integration of health service delivery where mental health components are included in the malaria prevention and treatment package, in order to reduce the burden of mental health adversity in children. Lastly, improvement of clinical practice through training of health professionals about the long-term outcomes of SM, SM with RMA, and the need to tackle the effects of malaria early on in a child’s life.
